# Annotations

**Published:** 1901-12-28

**Authors:** 


### Annotations.

### The Advantages of being an Invalid.

To the young who have before them the making of their careers, and in whom the instincts natural to youth are strong and imperative, the discovery that there is something wrong with their internal machinery, and that instead of taking their part in the fight of life they must paddle along gently as invalids, comes as a terrible revelation. To such the announcement of the existence of some form of disease which without threatening life threatens to take away what are then regarded as life's main pleasures, appears almost worse than a sentence of death itself, and for them we ought to feel every pity. But the verdict which cripples a man's efforts and limits his pleasures is not always a disadvantage, and it would be easy to point out many an example of the advantages of being an invalid, in the persons of elderly men who by dint of their invalidism are able at least to live on. So far as a man's own personal experience is concerned, he of necessity guides his life and arranges his work and his pleasures on the strength of an experience which is that of a younger man, and this is at the root of many catastrophes. When a man has passed his prime, something which shall with a firm hand limit his efforts, his work, his pleasures, his food, in accordance with what he may safely do rather than in accordance with what his own younger experience of himself tempts him to do, something which shall make him "a bit of an invalid" and break him off some of his habits, is not infrequently his salvation. Professor Osler has lately shown how important an influence in a man's life may be the discovery of a trace of albuminuria, and how the existence of such a malady as is thus indicated, while not in itself doing much harm, may, if properly dealt with, do much good, and one could point out quite a series of morbid states which are now and then brought to light "accidentally" as it is called—in the course of an examination for insurance, for example—the discovery of which, if received in proper spirit and followed up by proper precautions, may very clearly demonstrate the advantages of being an invalid.

### Susceptibility.

In a Blue Book just issued, many details are given concerning the prevalence of infectious disease in the Boer concentration camps. Among other things, it is pointed out how large an influence in producing a virulent type of measles has been the absence among the Boers of that immunity to future attacks which so often follows in those who suffer from this disease. The fact would seem to be that on many of the Boer farms during ordinary times so isolated has been the life, so rare the importation of infection, and so few the chances of its spreading from farm to farm that, so far as measles is concerned, we have had to do with a virgin population. As in Fiji, where years ago a vast epidemic of measles, till then unknown in the land, decimated the population, so in South Africa when new conditions were introduced, when the isolation of family from family on which their protection had depended was done away with, and when suddenly these susceptible people were massed together either on commando or in our concentration camps, measles assumed at once a virulent form. Of course, the evil has been aggravated in every way by insanitation, but when we note the frequency with which the disease has attacked adults, and even old people; and when we are told, as we are told, that even in ordinary times when measles happens to be introduced into the farms it often carries off a considerable portion of adults; and when we learn how extensively the disease has prevailed among the adult prisoners at St. Helena we may be quite sure that much of the gravity of the present outbreak is due to the fact that the mass of the population is unprotected by previous attacks. A question of some interest to ourselves arises from all this. In London we can hardly be said to suffer from epidemic measles, large as is the loss of life from that disease. Every year it affects a large number of children, and we can well believe that the reason why great epidemics do not take place is that the proportion of the population which is susceptible never rises high enough to support a grand outburst. Year after year, with monotonous regularity, a good year is followed by a bad year, a year's growth of susceptible children providing food for a new accession of the disease, and it is a matter of considerable moment to speculate what would happen were we to succeed by isolation in definitely holding the disease in check, say for half a dozen years—at the same time, of course, holding in check the immunity which follows the disease, and thus producing a vast population of highly susceptible children. That in ordinary times many lives would be saved seems very probable. But that we should have some fine epidemics no one can doubt.

### A Nose Moulded in Paraffin.

The surgeon who takes his vocation seriously is apt to feel that he is rather being made a tool of when he is asked to perform operations for purely æsthetic reasons. Still, happiness is often so completely destroyed by the possession of unpleasant personal peculiarities, and the increase of human happiness is so legitimate an object of human effort, that what is called cosmetic surgery must never be despised. One of the latest developments of this art is said to be the moulding of noses in paraffin which, if reports are to be believed, is now being practised in Vienna. In the deformity known as saddle-nose the most remarkable results are said to be obtained. The process consists in the subcutaneous injection of paraffin which, before it completely sets, is moulded into the desired shape. A warmed syringe is charged with the melted compound and the needle is inserted between the eyebrows, just above the root of the nose, and passed down to the lowest point of the excavation. Then the compound is injected into the subcutaneous tissue as the needle is slowly withdrawn. While it is still warm the paraffin under the skin is moulded into the proper shape and "there you are!" It is stated that practically no irritation results, either immediately or subsequently, and that cases have been observed for now over a year which show complete tolerance on the part of the tissues and in which a permanent correction of the deformity has been obtained. The compound made use of is a mixture of the solid with the soft forms of paraffin in such proportions as to produce a mixture having a melting-point of 41° C. Of course everything must be sterilise.

